# Differences in non-communicable diseases between women in prison and the general population in Brazil

**DOI:** 10.1038/s41598-023-46045-8

**Published:** 2023-10-31

**Authors:** Marto Leal, Ligia Kerr, Rosa Maria Salani Mota, Roberto da Justa Pires Neto, David Seal, Carl Kendall

**Affiliations:** 1 Department of Community Health, Federal University of Ceará, Rua Professor Costa Mendes, 1608-Bloco Didático, 5º Andar, Bairro Rodolfo Teófilo, Fortaleza, Ceará CEP: 60.430-140 Brazil; 2 Department of Statistics and Applied Mathematics, Federal University of Ceará, 100 Cinco Street-Bloco 910, Fortaleza, CE 60.355-636 Brazil; 3 Department of Global Community Health and Behavioral Sciences, Tulane University, 1440 Canal Street, New Orleans, LA 70112 USA

**Keywords:** Risk factors, Cardiovascular diseases

## Abstract

Women in prison have high risk for non-communicable diseases both in relation to men in prison and in relation to women in the general population. This study documented the health disparities related to diseases among women in prison and in the general female population in Brazil. Women in prisons (WP) < 30 years old had a prevalence of hypertension (PR = 4.5; 95% CI 3.4–6.1), cardiovascular disease (PR = 4.4; 95% CI 2.4–7.9) and asthma (PR = 3.0; 95% CI 2.3–3.8) higher than general female population in Brazil in the same age group. Women in prison > 50 years old also presented asthma prevalence (PR = 4.3; 95% CI 2.9–6.3) higher than the general female population in Brazil in the same age group. These women in prison, overwhelmingly young, could be mistaken for an elderly population in Brazil. Actively responding to early disease in these women can reduce overall health costs and improve health care for this population that may have limited access to health care outside of prison.

## Introduction

There has been a 53% increase in the number of women in prison (WP) during the last three decades, compared with a 19% increase among men^1^ in Brazil. WP increased 656% from the early 2000s to 2016 versus 293% among man in prison. More than 42,000 women are currently in prison in Brazil, representing a rate of 21.7 per 100 thousand inhabitants^2^.

Various forms of prejudice, human rights violations and income inequality play out in the Brazilian prison system and it is increasing under the new Federal government of Brazil^3,4^. This contributes to the fact that the prison experience has a strong potential to promote health disparities^5^. Consequently, WP have a high risk of health problems both in relation to men in prison and in relation to women in the general population (WGP)^6^. Furthermore, WP have a greater burden of noncommunicable diseases (NCDs) than the general population^7,8^, despite being younger.

Thus, the objective of this study was to document the health disparities in NCDs between women in prison and the general female population in Brazil.

## Methods

We compared two cross-sectional national surveys in Brazil. The first, Health of Women in Prison in Brazil (HWP), was conducted in 15 prisons in 8 Brazilian states and in the Federal District by the research team represented here by the authors. The second, the National Health Survey (NHS), was conducted by the Ministry of Health in Brazil. We confirm that all methods were carried out in accordance with relevant ethical and scientific guidelines.

### Women in prison

WP included any woman sentenced to prison in either a closed or semi-open regime (i.e., not house arrest) in one of our selected prisons. Women who were unable to participate due to poor health, considered dangerous by prison authorities, or who were not Portuguese speakers were excluded. All political-administrative regions in Brazil were included in the sample. First, two states with the largest female prison population in each region were chosen^9^. Next, we stratified prisons in Brazil into strata by size (75–150, 151–500 and over 500) and by location (metropolitan area vs rural area). Only facilities with seventy-five or more prisoners were included in our sample since they are required to have an on-site health center as needed for our study. These prisons constitute 4/5 of the total number of female prisons in Brazil. The resulting classification by location and size generated 24 strata, in the five administrative regions containing 10,668 women in prison. We selected one prison at random from each stratum. To calculate sample size, we used an estimated prevalence for sexually transmitted infection (STIs) of 30%. STIs are one of the diseases we explore in Health of Women in Prison in Brazil study, and was the most current available for each prison. Selecting a level of significance of 95% and power 80% we calculated an initial sample size of 1861 women in prison. Final sample selection occurred in the prisons, systematically selecting women from a daily list of prisoners provided by the prison authorities at each site. At the prison level we discovered a difference between the Federal Ministry of Justice count of the WP and actual numbers in the prison. Overall, there were 8.3% (n = 154) fewer women than reported in the official Federal report. Additionally, 10.5% refused or withdrew (n = 195). Logistical and on-site issues resulted in a further reduction of 9.9% (n = 185). The final sample was 1327 women. Audio Computer-Assisted Self-Interview (ACASI) was offered to each WP^10^. The project was described to each potential participant and the consent form read. Upon consent, the participant was handed a tablet and provided instruction on its use. Most respondents were uncomfortable using the tablet perhaps because of low levels of education and familiarity with the technology and preferred to be interviewed directly by our staff. A closed-ended questionnaire collecting sociodemographic, health, economic, personal prison history and experience were applied. Interviews were conducted in a private area, most often a room attached to the prison health center.

### Women in the general population

WGP was selected using three-stage cluster sampling^11^. In the first stage, the selection of the primary sampling units (census sectors or combination of sectors) was carried out by simple random sampling. In the second stage, a fixed number of permanent private households were randomly selected in each primary sampling unit (PSU) selected in the first stage. In the third stage, within each household, one resident (18 or over) was selected with equal probability, from a list of eligible residents constructed at the time of the interview. The sample identified 60,202 households. For this study, only data from households in which the respondent was female was used. The total sample was 31,845 women (52.9% of the total NHS sample). The NHS conducted by the Brazilian Ministry of Health is conducted periodically and recognized internationally. Details of sample and weighting can be found at https://www.pns.icict.fiocruz.br/.

### Statistical analyses

The prevalence of the variables of interest with 95% confidence intervals were estimated for both samples separately. The analyses were performed using the complex analysis module in STATA®v.15. The sample was weighted according to the sampling design, with the weight being the inverse of the product of the probabilities of the sampling units at each of the stages of the sample design. Initial data coding and cleaning was conducted in SPSS® v 20.0.

Self-reported prevalence of hypertension, other cardiovascular diseases, diabetes, hypercholesterolemia, asthma, cancer and stroke among WP and WPG was estimated. The health disparities between the groups were explored by calculating the prevalence ratio (PR) and confidence intervals (95%CI). Stratified and age-adjusted analyzes were then performed using Poisson regression. For all analyzes, the WGP group was considered as a reference. The analysis took age into account stratifying by the following age groups: < 30 years; 30–49 years; and ≥ 50 years. P-values < 0.05 were considered statistically significant. Chi square tests were used to compare the profile of the groups.

Approval of the research protocol: the Research Ethics Committee of the Federal University of Ceará Ethics Committee reviewed and approved the aims and procedures of this study (protocol n° 188,211). Informed consent: For consent to participate in the study, the participants signed an approved Free and Informed Consent Form that emphasized the voluntary nature of participation and lack of consequences for non-participation. Informed consent was obtained from all individual participants included in the WP study. The NSF followed consent procedures as described in project documents. All methods were carried out in accordance with relevant guidelines and regulations.

### Ethics declarations

The Research Ethics Committee of Federal University of Ceará Ethics Committee reviewed and approved the aims and procedures of this study (protocol n° 188.211). Informed consent was obtained from all individual participants included in the study.

## Results

A total of 1327 WP and 34,282 WGP were included in the study. Regarding age groups, 43.9% of WPs were < 30 years old versus 24.9% among WGPs. Approximately 15% of WPs versus 9% of WGP self-reported as black. Almost half of WPs and 40% of WPGs were illiterate or had incomplete elementary education (Table 1).

**Table 1:** Sociodemographic characteristics of women prisoners and the general population of Brazil.

Variables	WP		WGP		p-value
	N	%	N	%	
Age					
< 30 years	587	43.9	7.918	24.9	< 0.001
30–49 years	632	47.6	12.714	39.9	
≥ 50 years	113	8.5	11.212	35.2	
Total	1.327	100.0	31.845	100.0	
Race/ethnicity					
White	383	31.5	15.301	48.0	< 0.001
Black	199	15.3	2.933	9.2	
Yellow	34	2.4	327	1.0	
Mixed race	685	49.8	13.133	41.2	
Indigenous	17	1.0	149	0.5	
Total	1.318	100.0	31.845	100.0	
Education					
Illiterate/incomplete elementary school	660	48.4	12.141	38.1	< 0.001
Complete elementary school/incomplete high school	430	33.1	4.662	14.6	
Complete high school/incomplete college	217	17.3	10.608	33.3	
Complete college	17	1.2	4.434	13.9	
Total	1.324	100.0	31.845	100.0	
Marital status					
Single	597	43.5	12.781	40.1	< 0.001
Married/stable partner	601	46.6	13.453	42.2	
Separated/divorced	82	6.7	2.367	7.4	
Widow	44	3.2	3.243	10.2	
Total	1.325	100.0	31.845	100.0	
Has health insurance					
No	1.200	91.0	21.963	69.0	< 0.001
Yes	119	9.0	9.882	31.0	
Total	1.319	100.0	31.845	100.0	

*N* observed values, *%* weighted estimate.

WP < 30 years old had a prevalence of hypertension (PR = 4.5; 95% CI 3.4–6.1), cardiovascular disease (PR = 4.4; 95% CI 2.4–7.9) and asthma (PR = 3.0; 95% CI 2.3–3.8) higher than WGP in the same age group. WP between 30 – 49 years old had a prevalence of hypertension (PR = 1.7; 95% CI 1.4–2.0), cardiovascular disease (PR = 2.0; 95% CI 1.4–3.0) and asthma (PR = 3.3; 95% CI 2.6–4.0) higher than WGP in the same age group. WP > 50 years old also presented asthma prevalence (PR = 4.3; 95% CI 2.9–6.3) higher than WGP in the same age group. There were no statistical differences among age in relation to the prevalence of diabetes. WP between 30 and 49 years old presented cancer (PR = 2.1; 95% CI 1.2–3.7) and stroke (PR = 2.2. 95% CI 1.1–4.2) prevalence higher than WGP in the same age groups (Table 2).

**Table 2:** Prevalence ratio of different morbidities between WP and WGP adjusted by age group, Brazil, 2020.

Variables	WP (%)	WGP (%)	PR	95% CI	p-value
Perception of health status					
Regular/bad/very bad versus very good/good^1^					
< 30 years	34.9	21.9	1.59	1.38–1.83	< 0.001
30–49 years	40.8	32.7	1.24	1.12–1.39	< 0.001
> 50 years	57.4	54.1	1.06	0.89–1.26	0.507
Arterial hypertension					
< 30 years	13.0	2.9	4.54	3.36–6.12	< 0.001
30–49 years	29.0	16.8	1.72	1.48–2.00	< 0.001
> 50 years	56.2	48.4	1.16	0.97–1.39	0.106
Other cardiovascular diseases					
< 30 years	5.1	1.1	4.35	2.41–7.87	< 0.001
30–49 years	7.0	3.3	2.08	1.42–3.05	< 0.001
> 50 years	11.8	10.2	1.15	0.63–2.11	0.639
Diabetes					
< 30 years	0.6	0.7	0.86	0.23–3.19	0.821
30–49 years	3.3	4.0	0.82	0.50–1.36	0.455
> 50 years	14.2	17.2	0.83	0.52–1.32	0.432
Hypercholesterolemia					
< 30 years	1.6	3.8	0.43	0.22–0.83	0.011
30–49 years	7.4	12.8	0.27	0.40–0.82	0.002
> 50 years	24.4	29.4	0.83	0.57–1.20	0.331
Asthma					
< 30 years	18.7	6.3	2.97	2.32–3.79	< 0.001
30–49 years	20.7	6.2	3.31	2.68–4.08	< 0.001
> 50 years	23.6	5.4	4.29	2.92–6.31	< 0.001
Cancer					
< 30 years	1.3	0.7	1.81	0.54–6.04	0.334
30–49 years	3.8	1.7	2.17	1.24–3.79	0.006
> 50 years	3.5	6.1	0.58	0.18–1.85	0.360
Stroke					
< 30 years	0.4	0.2	1.98	0.40–9.82	0.399
30–49 years	2.5	1.1	2.20	1.13–4.25	0.019
> 50 years	3.7	5.2	0.70	0.26–1.89	0.490

% weighted estimate.

^1^Reference categories.

## Discussion

This study showed important disparities between WP and WGP regarding NCDs. The younger WP not only perceive their health as worse than WGP at the same age but also have a higher prevalence of NCDs. WP are characterized by a lower level of schooling compared to the WGP^12^. They suffer the burdens of the intersection of multiple vulnerabilities, as they are poor, female, black, and incarcerated^6^. These women bring with them into prison a background and context that favors these diseases^13^. This finding, combined with the high prevalence of risk factors for degenerative NCDs^14^, such as obesity^8,15,16^, alcohol and other drug use^17,18^ and unhealthy diet^19,20^, makes the prison system an environment of high demand for services that are not commonly demanded by a young population, for example, antihypertensive therapy.

Self-report of asthma, hypertension and cardiovascular disease were higher among WP. A study comparing the prevalence of NCDs among the general prison population in the United States identified similar results for asthma (PR = 1.31; 95% CI 1.19–1.45) even adjusted for socio-demographic variables and alcohol consumption^20^. The association between respiratory disease and prison could also be explained by the poor environment observed inside the prisons^7^.

Interestingly, both cancer and stroke were more frequent in the WP than in the general population for the age group of 30–49 years. This stage of life is characterized by a time of greater responsibilities for family, especially for children. Being a prisoner means that many women stop providing family support, since many are single mothers and the only source of income, as found in both our study and the literature^21^. This disruption may favor the appearance of several health problems, including cancers and stroke^22^. Also, the lack of prevention programs in prison for the most common female cancers, hypertension and cardiovascular diseases that precede stroke should play a role.

We did not find differences in diabetes prevalence between WP and WGP. The availability of sugar-rich foods, such as sweets and soft drinks is limited within prisons, which might serve a protective function.

This study has some limitations. Prison populations are considered a vulnerable population for ethical reasons, so access of researchers to this population faces a series of regulatory hurdles. Even with judicial authorization, some facilities refused to participate, pushed back dates or otherwise did not facilitate the access of the research team to the prison. The consequences of these delays for our limited budget, effectively removed several sites from the study and reduced our sample size. Although we measured blood pressure and collected blood, we also collected self-report of diseases and conditions to compare to the National Health Survey, which just collects self-report data. Self-report data suffers from well-known limitations, and it is possible that the prevalence of NCDs have been underestimated in both studies.

Despite these limitations, our results suggest that the female prison population, although younger than the general female population, has an illness profile similar to older women in the general population. These results can be used as a guide by stakeholders to allocate resources for early identification and treatment of conditions that have consequences for the health system and the economy. These diseases not only produce direct health consequences, but also a cascade of social and societal effects. Actively responding to disease in prisoners under state responsibility is not only cost-effective common sense to reduce health disparities, but can also reduce overall health costs and improve the opportunities of prisoners and society post-prison. It is also a matter of respect for human rights.

## Data Availability

All data analyzed in this study is open access and can be obtained from Ligia Kerr’s email: ligiakerr@gmail.com.
